# Improved Efficiency of Intraventricular Blood Flow Transit Under Cardiac Stress: A 4D Flow Dobutamine CMR Study

**DOI:** 10.3389/fcvm.2020.581495

**Published:** 2020-11-25

**Authors:** Jonathan Sundin, Jan Engvall, Eva Nylander, Tino Ebbers, Ann F. Bolger, Carl-Johan Carlhäll

**Affiliations:** ^1^Unit of Cardiovascular Sciences and Center for Medical Image Science and Visualization, Department of Health, Medicine and Caring Sciences, Linköping University, Linköping, Sweden; ^2^Department of Clinical Physiology in Linköping, Department of Health, Medicine and Caring Sciences, Linköping University, Linköping, Sweden; ^3^Department of Medicine, University of California, San Francisco, San Francisco, CA, United States

**Keywords:** stress cardiovascular magnetic resonance, 4D flow CMR, left ventricle, hemodynamics, flow physiology, flow patterns, dobutamine stress

## Abstract

**Introduction:** The effects of heart rate, inotropy, and lusitropy on multidimensional flow patterns and energetics within the human heart remain undefined. Recently, reduced volume and end-diastolic kinetic energy (KE) of the portion of left ventricular (LV) inflow passing directly to outflow, Direct flow (DF), have been shown to reflect inefficient LV pumping and to be a marker of LV dysfunction in heart failure patients. In this study, we hypothesized that increasing heart rate, inotropy, and lusitropy would result in an increased efficiency of intraventricular blood flow transit. Therefore, we sought to investigate LV 4D blood flow patterns and energetics with dobutamine infusion.

**Methods:** 4D flow and morphological cardiovascular magnetic resonance (CMR) data were acquired in twelve healthy subjects: at rest and with dobutamine infusion to achieve a target heart rate ~60% higher than the resting heart rate. A previously validated method was used for flow analysis: pathlines were emitted from the end-diastolic (ED) LV blood volume and traced forward and backward in time to separate four functional LV flow components. For each flow component, KE/mL blood volume at ED was calculated.

**Results:** With dobutamine infusion there was an increase in heart rate (64%, *p* < 0.001), systolic blood pressure (*p* = 0.02) and stroke volume (*p* = 0.01). Of the 4D flow parameters, the most efficient flow component (DF), increased its proportion of EDV (*p* < 0.001). The EDV proportion of Residual volume, the blood residing in the ventricle over at least two cardiac cycles, decreased (*p* < 0.001). The KE/mL at ED for all flow components increased (*p* < 0.001). DF had the largest absolute and relative increase while Residual volume had the smallest absolute and relative increase.

**Conclusions:** This study demonstrates that it is feasible to compare 4D flow patterns within the normal human heart at rest and with stress. At higher heart rate, inotropy and lusitropy, elicited by dobutamine infusion, the efficiency of intraventricular blood flow transit improves, as quantified by an increased relative volume and pre-systolic KE of the most efficient DF component of the LV volume. The change in these markers may allow a novel assessment of LV function and LV dysfunction over a range of stress.

## Introduction

The heart generates blood flow through the cardiovascular system in order to supply the body's tissues with blood (1). The dynamic flow through the heart itself is yet to be completely characterized, prompting a growing interest in multidimensional intracardiac blood flow as studied with 4D flow cardiovascular magnetic resonance (CMR) (2, 3). This versatile and promising technique enables visualization and quantification of multidimensional blood flow patterns and energetics in the left ventricle (LV) (4–6), right ventricle (RV) (5, 7), left atrium (LA) (8, 9), and right atrium (RA) (9). It has been performed on normal hearts and on hearts of patients with acquired and congenital diseases.

4D flow CMR has been used to characterize intraventricular blood flow by separating the flow into four components (6, 10). One of these flow components is Direct flow (DF), which consists of blood entering the LV in diastole and being ejected in the ensuing systole. In normal hearts, DF has an efficient transit through the LV and possesses higher kinetic energy (KE) at end-diastole (ED) compared to the other three components (6, 11). The KE at ED for DF implies how well the energy of the LV inflow is preserved. A reduced DF has been used as a marker for ventricular dysfunction (10, 12).

At rest, the contribution of the KE possessed by the blood at ED to the total external systolic work performed by the heart is small. This KE at ED can have other beneficial effects, however, such as priming the aortic valve for ejection, and serving as a flow-based marker for subtle ventricular dysfunction (12, 13). Furthermore, preserving KE during ventricular diastole has been proposed to play an important role for diastolic-systolic coupling and systolic ejection with higher heart rates (HR) and cardiac output (CO) (14, 15). Previous 4D flow CMR studies of healthy humans have been conducted at rest, however, and the effects of heart rate, inotropy and lusitropy on multidimensional flow patterns and energetics within the healthy human heart remain undefined.

Pharmacological and physical stress tests are performed clinically to expose abnormalities in cardiac function. One of the compounds used for this is dobutamine, which is a synthetic catecholamine which exerts its effect by stimulating adrenoreceptors. Physiologically the effects of dobutamine can include an increase in inotropy (contractility), chronotropy (heart rate), lusitropy (relaxation), and some decrease in total peripheral vascular resistance (16, 17).

In this study, we sought to investigate LV 4D blood flow patterns and energetics with dobutamine infusion. We hypothesized that increasing heart rate, inotropy and lusitropy would result in an increased efficiency of intraventricular blood flow transit.

## Methods

### Study Population

The study population consisted of twelve subjects including eight women. Inclusion criteria were as follows: no history of cardiovascular disease, no medication for cardiovascular disease and a normal physical examination. The exclusion criteria were: absence of normal ventricular size, wall thickness or wall motion based on balanced steady-state free precession (bSSFP) CMR data at rest, more than moderate arterial hypertension, acute coronary artery disease, severe aortic stenosis, and hypertrophic obstructive cardiomyopathy. The study was approved by the Regional Ethical Review Board in Linköping and complies with the Declaration of Helsinki. All subjects provided written informed consent prior to participation in the study.

### Study Plan

**Figure 1:**
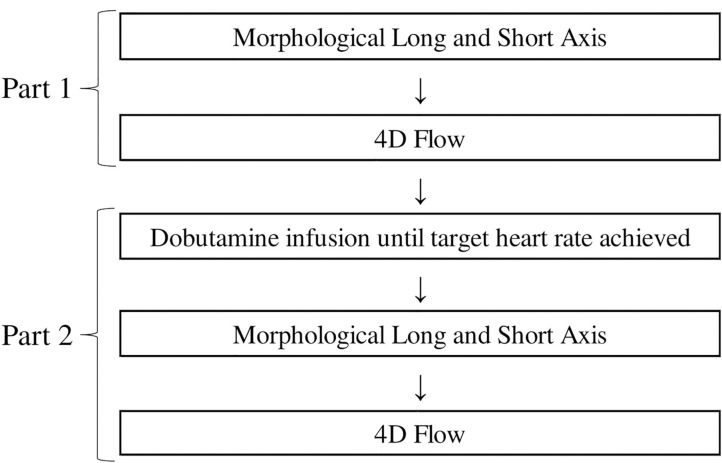
The CMR protocol. Part 1 is performed at rest and Part 2 during dobutamine infusion.

The subjects underwent a CMR protocol with two different parts, one part at rest (baseline) and a second part during the infusion of dobutamine (Figure 1). Dobutamine was administered intravenously starting at a dose between 5 and 10 μg/kg/min, and the dose was increased or decreased in intervals of approximately 2 min depending on the observed effect on the subject's heart rate and arterial pressure. The target heart rate was 60% higher than the subject's resting heart rate. Heart rate was monitored continuously throughout the study. The blood pressures were measured at rest and during the administration of dobutamine, approximately every second minute, using a cuff sphygmomanometer. The dobutamine infusion was maintained until the CMR data in the second part were acquired. Criteria for interrupting the infusion were achievement of the maximal systolic blood pressure (220 mmHg) or any discomfort considered to be due to dobutamine. These conditions did not occur in any of the subjects included in the study.

### CMR Data Acquisition

CMR scans were performed using a 3T Philips Ingenia scanner (Phillips Healthcare, Best, the Netherlands) to acquire morphological bSSFP images and 3D cine phase-contrast (4D flow) velocity data of the left heart. Morphological images were acquired during end-expiratory breath holds. Both scans (rest and dobutamine) included three-chamber long axis images and a stack of short axis images. Also, at rest four-chamber long axis images were acquired (Figure 1). Scan parameters were: echo time = 1.4 ms, repetition time = 2.8 ms, flip angle = 45°, slice thickness = 8 mm. The bSSFP images were reconstructed into 30 time frames. The short-axis images had an acquired resolution of 0.9 × 0.9 mm^2^ and the long-axis images acquired resolution was 0.83 × 0.83 mm^2^.

The 4D flow data were acquired during free breathing using a navigator-gated gradient-echo pulse-sequence with interleaved three-directional flow-encoding and retrospective vector cardiogram controlled cardiac gating (18). Scan parameters were: velocity encoding (VENC) = 140 cm/s, flip angle = 5°, echo time = 2.6 ms, repetition time = 5.2 ms, parallel imaging (SENSE) speed up factors = 3 (AP direction), 1.6 (RL direction), k-space segmentation factor = 2, elliptical k-space acquisition. The spatial resolution was 2.8 × 2.8 × 2.8 mm^3^ and the temporal resolution approximately 40 ms. This resulted in a scan time of approximately 7–8 and 10–15 min excluding and including navigator efficiency, respectively, for a heart rate of approximately 60 bpm.

### Data Analysis

The acquired 4D flow data were post-processed and analyzed using a previously validated method for intraventricular blood flow (18). The end-diastolic volume (EDV) and the end-systolic volume (ESV) of the LV were segmented in the short-axis stack with the long-axis images for guidance. The segmentations were performed using the freely available segmentation software Segment version 1.9 (Medviso, Lund, Sweden) (19).

**Figure 2:**
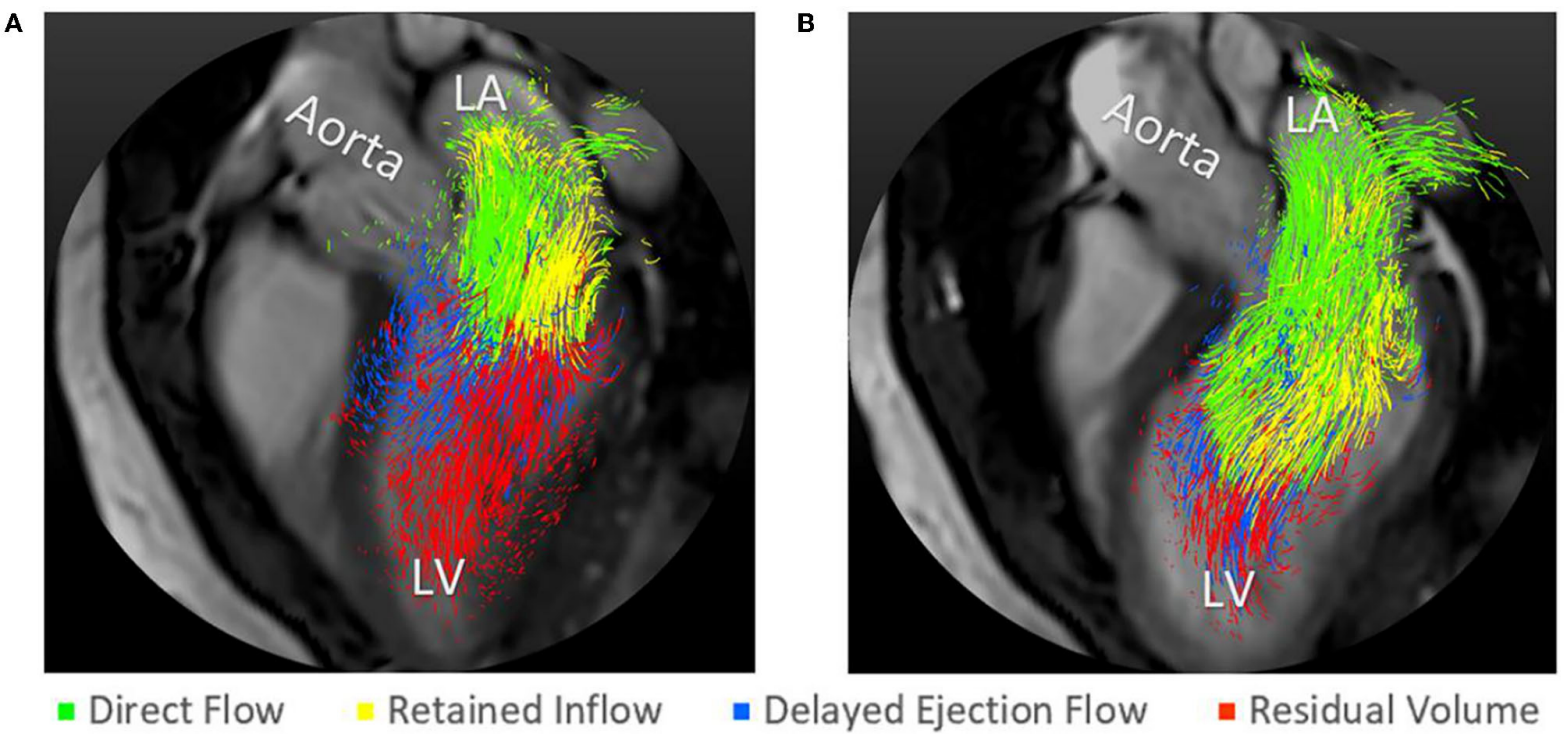
Pathline visualization of the four LV flow components in early diastole at rest **(A)** and with dobutamine infusion **(B)**.

The EDV and ESV segmentations were resampled to match the 4D flow data resolution. Pathlines were emitted from each voxel of the EDV and traced forward and backward in time until ESV, covering the entire heart cycle. The EDV was separated into four functional flow components, based on the pathlines' route through the LV, as previously defined by Bolger et al. (6) (Figure 2). The four components included *Direct flow*, the blood that enters the LV in diastole and leaves it during systole in the analyzed heart beat; *Retained inflow* (RI), the blood that enters the LV in diastole but does not leave it during systole of the analyzed heart beat; *Delayed ejection flow* (DEF), the blood that starts out in the LV, and resides there during diastole and leaves it during systole in the analyzed heart beat; and *Residual volume* (RV) the blood that resides in the LV for at least two cardiac cycles. All datasets were subjected to quality control: LV inflow and outflow were compared for each subject, an inflow-outflow discrepancy of >15% resulted in exclusion. Pathlines were visually inspected using EnSight Standard version 10.0.3(b) (EnSight, CEI Inc, Research Triangle Park, NC, USA); inconsistencies indicating poor data quality resulted in exclusion. bSSFP long- and short-axis images were inspected for aortic and mitral regurgitation. The KE of the volume of each flow component was calculated over the cardiac cycle based on the volume of blood represented by each pathline (*V*), its velocity (*v*) over the cardiac cycle, and the density of blood (ρ = 1060 kg/m^3^), according to the following equation (10):

$$\begin{array}{l} KE=\frac{\rho Vv^{2}}{2} \end{array}$$

### Statistical Evaluation

The Kolmogorov–Smirnov test was used to assess if the data were normally distributed. For the normally distributed data, *t-*tests for paired samples were performed. In the cases when the data wasn't normally distributed, a Wilcoxon signed-rank test was performed. Result are given as group mean ± SD and a *P* < 0.05 was considered significant. Statistical analyses were performed using Statistica v.10 (Statsoft, Tulsa, OK). Assessment of inter- and intra-observer variability was performed with intra-class correlation coefficient (ICC) using SPSS v.26 (IBM Corp, 2019).

## Results

No complications were observed due to the infusion of dobutamine. The peak dose of infused dobutamine for all subjects was 22 ± 6 μg/kg/min (range 15–30 μg/kg/min).

Demographic data for the twelve subjects are shown in Table 1. With the infusion of dobutamine heart rate increased from 66 ± 9 to 108 ± 13 bpm (*p* < 0.001), an increase of 64%, systolic blood pressure increased from 118 ± 13 to 135 ± 15 mmHg (*p* = 0.02) while diastolic blood pressure had no significant change, 68 ± 9 to 63 ± 8 mmHg (*p* = 0.25). The left ventricular volumes changed with the infusion: LVEDV decreased from 153 ± 28 to 139 ± 35 mL (*p* = 0.004), LVESV decreased from 65 ± 17 to 36 ± 13 mL (*p* < 0.001), resulting in an increase of stroke volume from 88 ± 13 to 102 ± 25 mL (*p* = 0.01). Ejection fraction increased from 58 ± 5 to 74 ± 5% (*p* < 0.001) and cardiac output increased from 5.8 ± 1.0 to 11.0 ± 2.4 l/min (*p* < 0.001). All twelve subjects had an increase in heart rate and cardiac output, and all but two had an increase in systolic blood pressure and stroke volume.

**Table 1:** Demographic and clinical parameters.

	**Rest**	**Dobutamine**	***P*-value**
Age (years)	33 ± 13		
Gender (f/m)	8/4		
Height (cm)	172 ± 8		
Weight (kg)	68 ± 8		
HR (bpm)	66 ± 9	108 ± 13	<0.001
BP systolic (mmHg)	118 ± 13	135 ± 15	0.021
BP diastolic (mmHg)	68 ± 9	63 ± 8	0.251
LVEDV (mL)	153 ± 28	139 ± 35	0.004
LVESV (mL)	65 ± 17	36 ± 13	<0.001
LVEF (%)	58 ± 5	74 ± 5	<0.001
LVSV (mL)	88 ± 13	102 ± 25	0.010
CO (L/min)	5.8 ± 1.0	11.0 ± 2.4	<0.001

*HR, heart rate; BP, blood pressure; LV, left ventricle; EDV, end-diastolic volume; ESV, end-systolic volume; EF, ejection fraction; SV, stroke volume; CO, cardiac output*.

**Figure 3:**
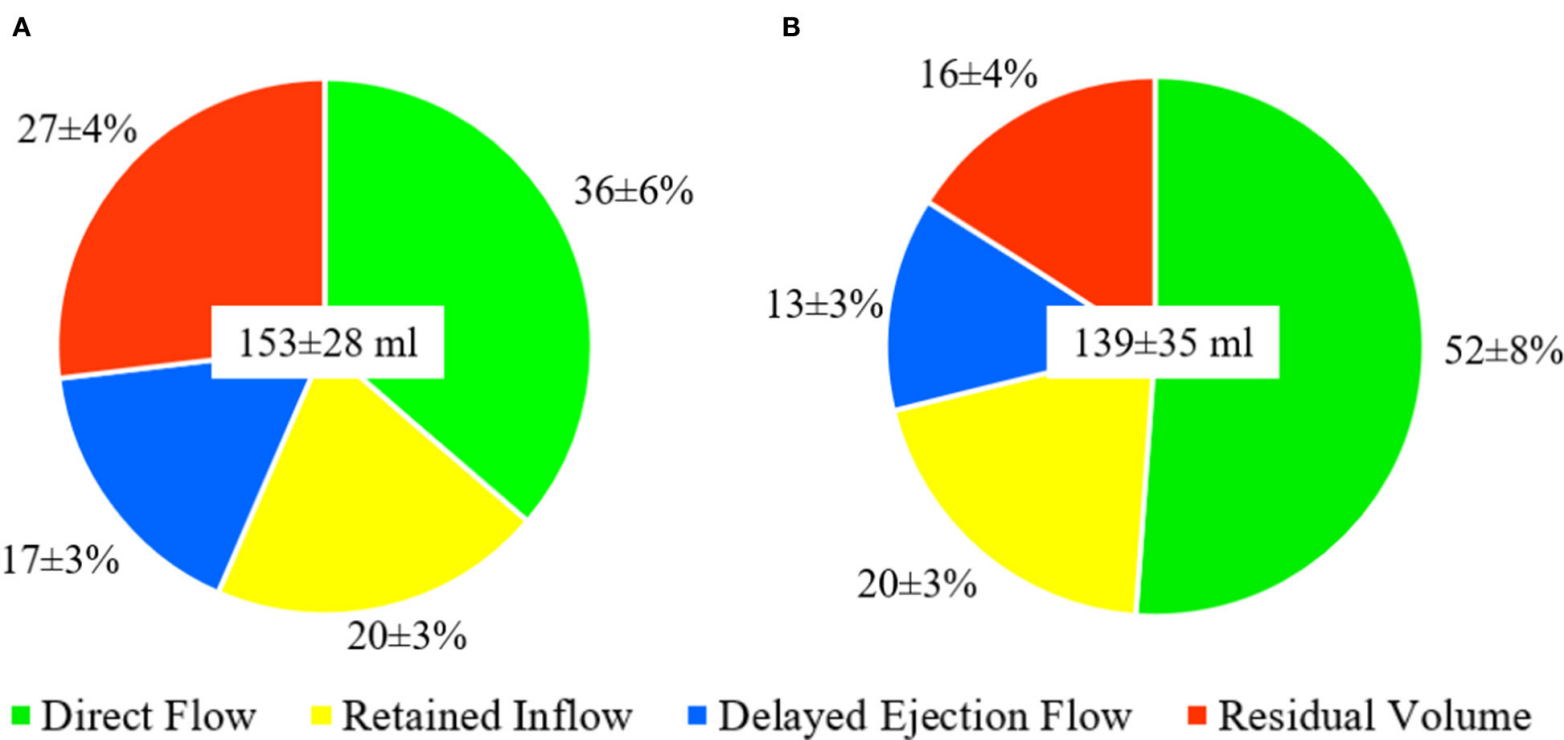
Distribution of the flow components presented as mean ± SD at rest **(A)** and with dobutamine infusion **(B)**. LV end-diastolic volume presented as mean ± SD mL in the center of the circles.

**Figure 4:**
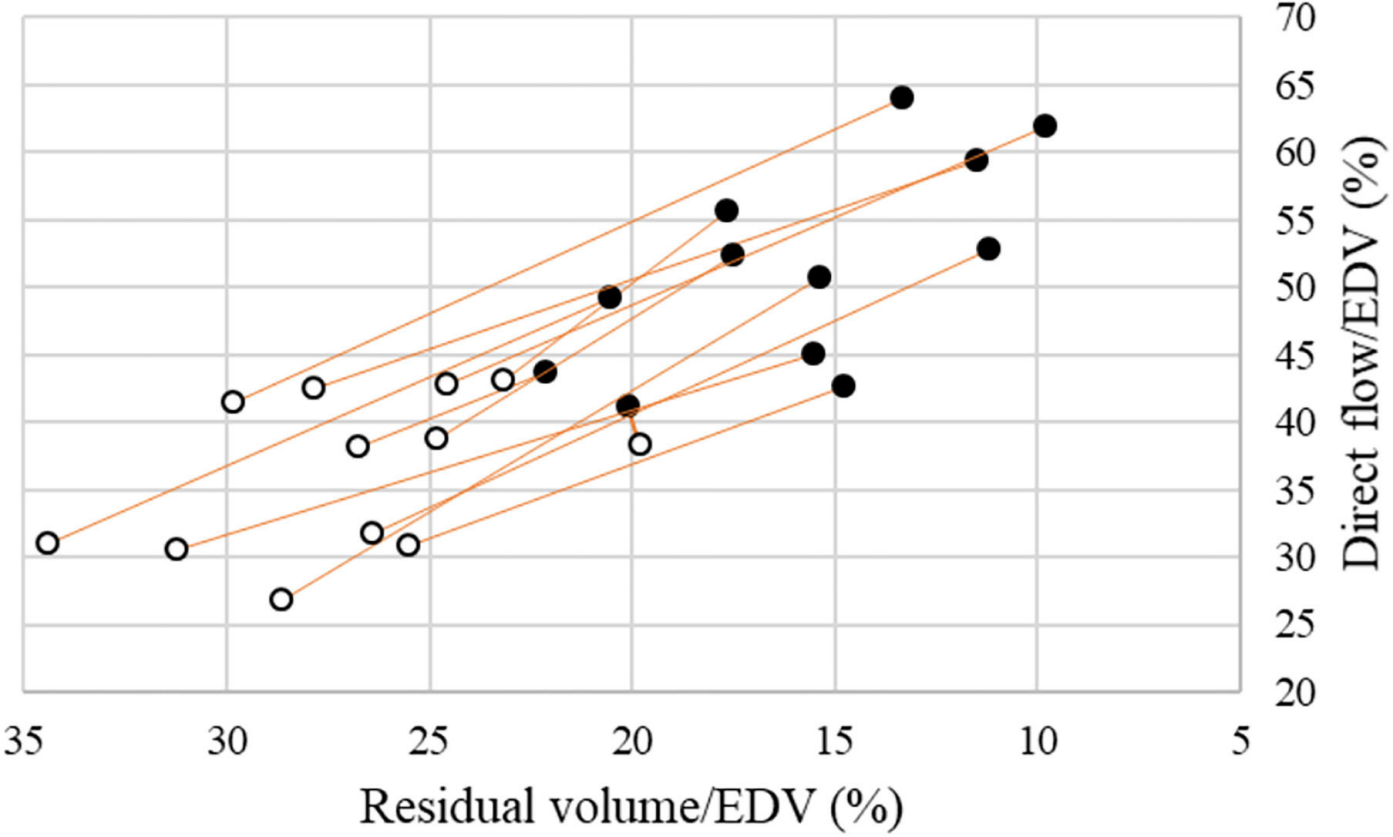
Volume proportion of EDV for Direct flow (Y-axis) and Residual volume (X-axis) at rest (white circles) and with dobutamine infusion (black circles) in the healthy subjects.

The 4D flow parameters are displayed in Table 2. In terms of the components‘ volume in proportion to the EDV, DF increased from 36 ± 6 to 52 ± 8% (*p* < 0.001), RI had no significant change from 20 ± 4 to 20 ± 3 (*p* = 0.43), DEF decreased from 17 ± 3 to 13 ± 3% (*p* = <0.001) and RV decreased from 27 ± 4 to 16 ± 4% (*p* < 0.001) (Figure 3). The variation of the relative volume of DF and RV at rest and with dobutamine infusion for each individual is shown in Figure 4.

**Table 2:** 4D flow parameters.

**Volume/EDV (%)**	**Rest**	**Dobutamine**	***P*-value**
DF	36 ± 6	52 ± 8	<0.001
RI	20 ± 3	20 ± 3	0.429
DEF	17 ± 3	13 ± 3	<0.001
RV	27 ± 4	16 ± 4	<0.001
**KE/volume at ED (μJ/mL)**			
DF	7.7 ± 3.0	21.0 ± 5.4	<0.001
RI	3.7 ± 1.4	9.6 ± 3.1	<0.001
DEF	5.8 ± 2.5	13.6 ± 6.0	<0.001
RV	1.5 ± 0.5	2.8 ± 1.0	<0.001

*EDV, end diastolic volume; DF, direct flow—blood that both enters and leaves the LV within one cardiac cycle; KE_ED_, kinetic energy at end-diastole; RI, retained inflow—blood that enters but does not leave the LV within one cardiac cycle; DEF, delayed ejection flow—blood that leaves but does not enter the LV within one cardiac cycle; RV, residual volume—blood that resides within the LV for at least two cardiac cycles*.

**Figure 5:**
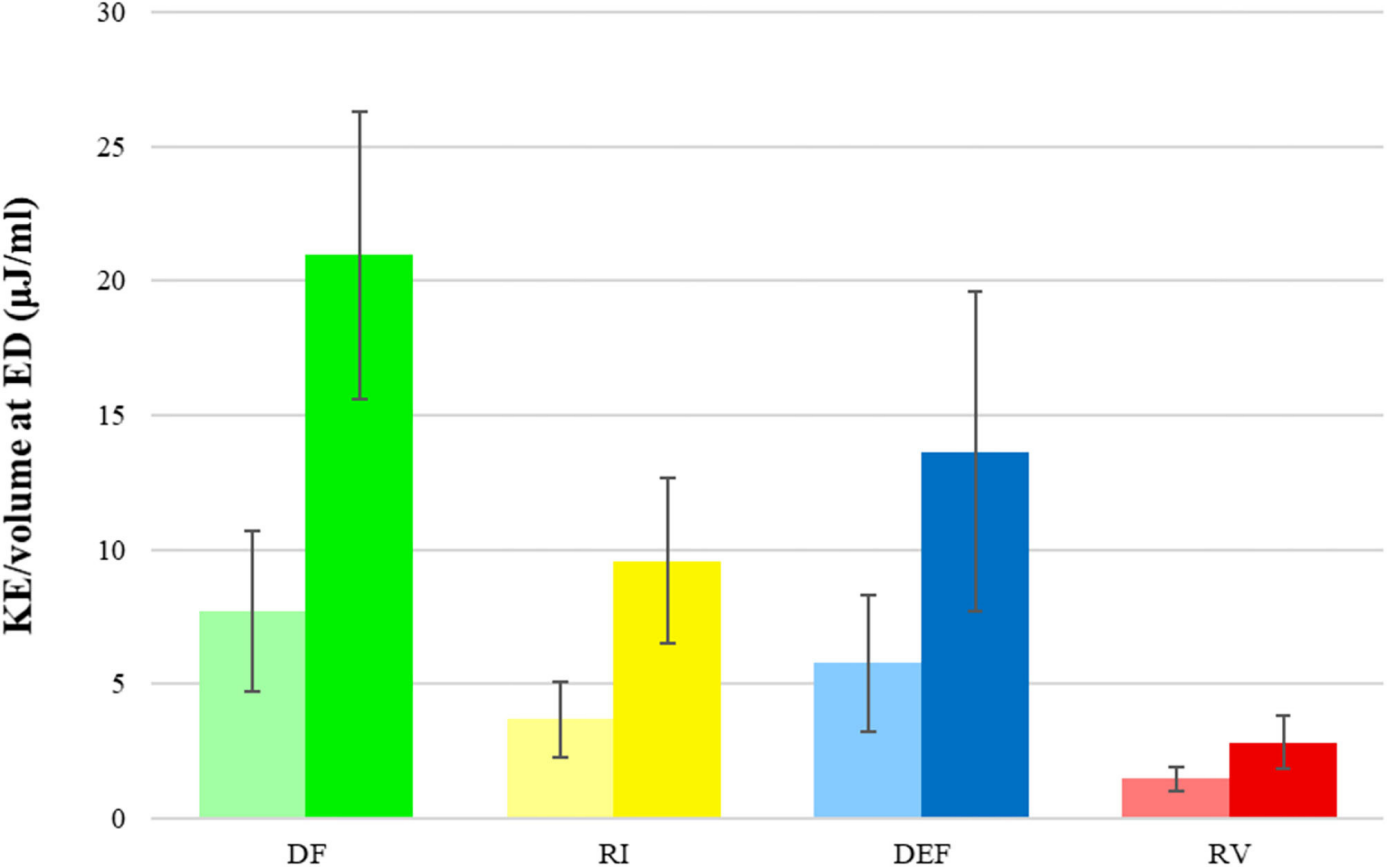
KE/volume (μJ/mL) for each flow component. Graphs in bright color (left) represent values at rest and graphs in dark color (right) represent values with dobutamine infusion. DF, direct flow; RI, retained inflow; DEF, delayed ejection flow; RV, residual volume.

In terms of the components‘ KE/volume at ED, the values for all the four components increased, for DF from 7.7 ± 3.0 to 21.0 ± 5.4 μJ/mL (*p* < 0.001), for RI from 3.7 ± 1.4 to 9.6 ± 3.1 μJ/mL (*p* < 0.001), for DEF from 5.8 ± 2.5 to 13.6 ± 6.0 μJ/mL (*p* < 0.001), and for RV from 1.5 ± 0.5 to 2.8 ± 1.0 μJ/mL (*p* < 0.001) (Table 2 and Figure 5).

**Figure 6:**
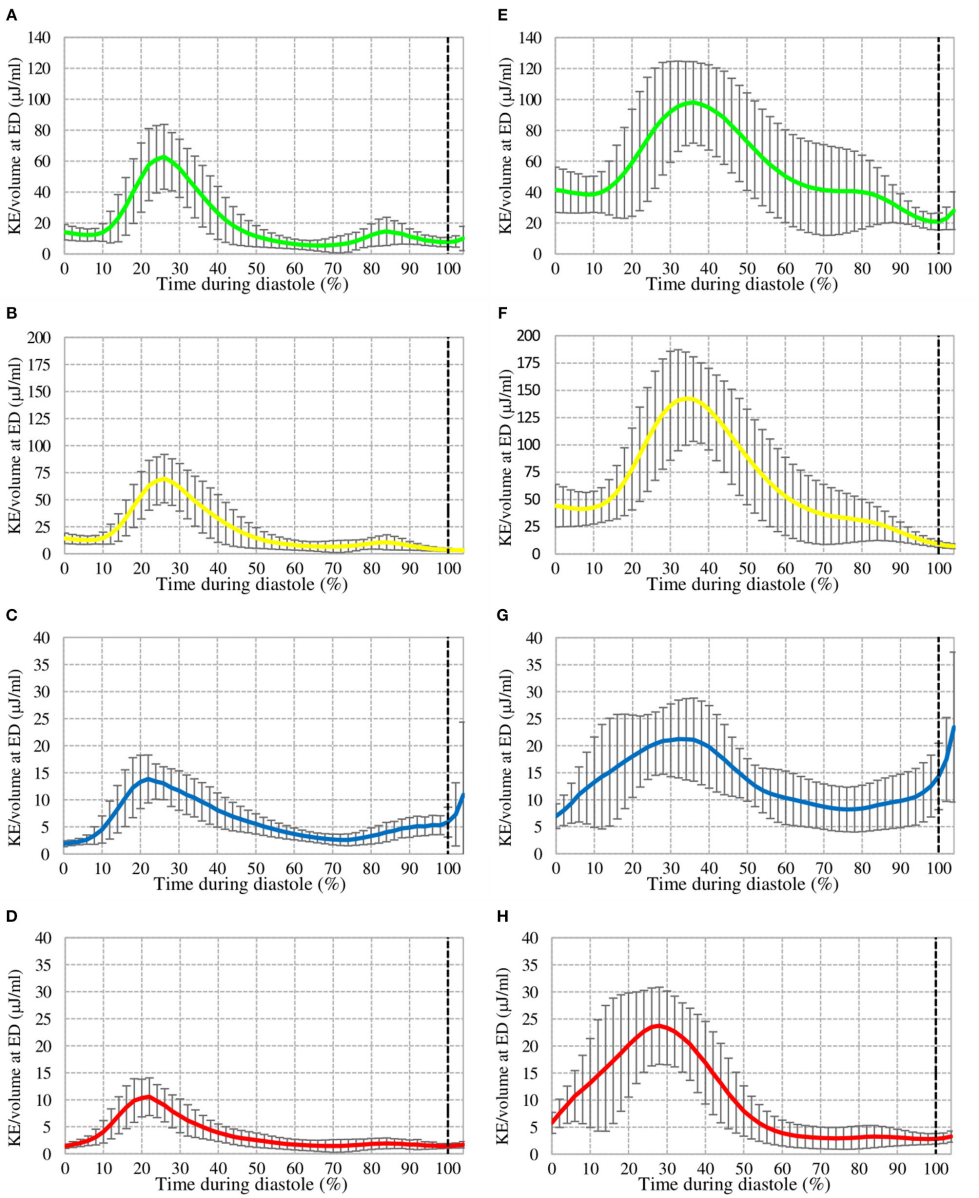
KE over diastole at rest (left) and with dobutamine infusion (right) for Direct Flow **(A,E)**, Retained Inflow **(B,F)**, Delayed Ejection Flow **(C,G)**, and Residual Volume **(D,H)**. The dotted line represents End-Diastole.

For KE/volume over diastole at rest, an early peak during the E-wave was seen for all components as well as a second, small peak late in diastole during the A-wave, which is substantially smaller for all components and almost non-existent for RV. The LV inflow components DF and RI have values for the early peak in the range of approximately 40–90 μJ/mL. The non-LV inflow components RV and DEF have an early peak in the range of 5–20 μJ/mL. With the dobutamine infusion there is a general increase of KE/mL in all flow components over the entire diastole, approximately 70–190 μJ/mL for the LV inflow components and 15–30 μj/mL for the non-LV inflow components at early filling. Also, the temporal pattern of the DF and RI graphs changed. With a shorter duration of diastole, the early filling phase appears relatively later in diastole, and the KE is maintained to a higher degree during diastasis (Figure 6).

Intra-observer variability for the LV flow components with dobutamine was excellent (ICC = 0.99) and inter-observer variability for the LV flow components with dobutamine was good to excellent (ICC = 0.80–0.91).

No left sided valve regurgitation was visualized at rest. At stress, trace regurgitation was present in four subjects, two with mitral regurgitation and two with aortic regurgitation.

## Discussion

In this study dobutamine was infused in healthy subjects to investigate the impact of heart rate, inotropic- and lusitropic state on multidimensional flow patterns and energetics within the LV. With dobutamine stress, the relative volume and pre-systolic KE of blood entering the LV and passing directly to ensuing ejection (Direct flow component) increased, and the relative volume of re-circulating blood (Residual volume component) decreased.

In our study a dobutamine peak dose of 22 ± 6 μg/kg/min, resulted in a 64% increase in HR, a 14% increase in systolic BP, a 16% increase in SV and a 90% increase in CO. Mandapaka et al. (20) administered a dobutamine and atropine dose which resulted in a 108% increase in HR, a 11.5% increase in systolic BP, a 13% increase in SV and a 134% increase in CO. Schuster et al. (21) administrated a dobutamine dose of 20 μg/kg/min that resulted in a 69% increase in heart rate, a 13% increase in SV and a 91% increase in CO. The results obtained in our study are in line with the results in these studies (20, 21). Two of the subjects had a reduction in systolic BP (both 19 mmHg). These two were the oldest of the subjects (56 and 57 years) and also showed the largest reduction in diastolic BP. These hypotensive responses can be due to a disproportionate decrease in systemic vascular resistance in the presence of a normal increase in cardiac output, an effect that has been reported previously in older subjects (22).

The DF component has a direct and efficient transit through the LV cavity. In normal hearts at rest, the inflowing KE of the DF blood is preserved to a certain extent at ED, and in particular for the late diastolic inflow (11). Dobutamine increases the inotropy of the LV which increases the ejection fraction and decreases the LV end-systolic volume. Accordingly, an increase in the DF proportion of EDV as well as a decrease in the RV proportion of EDV can be expected.

Kilner et al. (14) proposed that the exercising heart maintains the flow of the blood entering the ventricles by redirection and enhancement of momentum. While exercise is not entirely simulated with dobutamine, the KE of DF with dobutamine compared to rest (Figures 6A,E) is maintained to a higher degree and could be explained by the reasons stated by Kilner et al.

At ED, the KE/mL for each flow component was increased, and in particular for the efficient DF which had highest absolute increase as well as the highest relative increase of KE/mL at ED. This might be expected as with an increased heart rate a similar stroke volume is entering and prepared for ejection during a shorter diastolic filling phase.

LV blood flow patterns demonstrated with 4D flow CMR have recently been investigated at rest and with dobutamine stress in pigs (23). Interestingly, Cesarovic et al. (23) studied the relative amount of DF, and this parameter increased from 43 to 53% with an increase in heart rate from 82 to 124 bpm. This 23% increase in DF is smaller than the DF increase in our study (44%), and could be explained by a smaller increase in heart rate (51 vs. 64%) or by the different pathline method which they applied.

In a recent study by Kamphuis et al. (24), ventricular 4D flow was studied in ten Fontan patients at rest and with dobutamine stress. HR increased from approximately 84–111 bpm (33%) and stroke volume from 81 to 92 mL. The average KE of the total LV blood volume during both diastole and systole increased significantly with stress. Further, the increase in average KE of the total LV blood volume (rest-stress difference) showed a significant inverse correlation to maximum oxygen uptake (VO_2_) max measure with cardiopulmonary exercise testing (*r* = −0.83, *P* = 0.003). In our study VO_2_ max was not assessed, however our hypothesis is that the KE of the LV DF flow component would show a positive correlation and the KE of the LV RV flow component would show a negative correlation to VO_2_ max.

A previous study of the KE of the total LV blood volume over the entire cardiac cycle yielded similar values of KE at ED at rest to those which we acquired (5). In that study, a model was used to estimated systolic KE's contribution to stroke work during exercise. Assuming an exercise-induced HR of 160, a 57-fold increase of KE's contribution was predicted and a 7-fold increase in potential energy, increasing the contribution of KE to the stroke work from 0.3 to 2.9%. These results suggest that KE and its importance increases progressively with higher heart rates. In their model, the heart rate increased by about 160% compared to the 64% in this study, and the increase in blood pressure was more substantial as well.

The findings from the current study have several potential clinical applications. Remodeling and dysfunction of the LV is progressive and can be present before any clinical manifestations appear (25). Reduced DF relative volume and KE at ED have been suggested to be promising markers for LV dysfunction at rest by indicating altered transit of LV flow (10). The response of these flow-based parameters to dobutamine- induced stress on the heart may expand its usefulness in the assessment of subclinical and subtle LV impairment. Moreover, these novel parameters of LV flow efficiency may also assist in defining the target heart rate in heart failure (HF) patients (26). Heart rate control is one of the guideline-recommended treatment goals for HF patients (27). However, there is a lack of functional methods and parameters that can add to our understanding and definition of target heart rate in HF patients both with and without reduced ejection fraction. Furthermore, analysis of the stress-induced changes in the LV residual volume may contribute new aspects to the risk assessment for LV thrombus formation in HF patients by exposing blood flow abnormalities not present at resting conditions (28).

## Limitations

The study was performed on a relatively small cohort of healthy subjects and should be considered a feasibility study investigating multidimensional intracardiac blood flow with dobutamine stress. Future studies will focus on subjects with different ages, physical training volumes, and cardiac pathologies, which will clarify the clinical value of the approach. The results of the current study are limited to a supine body position and a completely still body. bSSFP and 4D flow data were acquired directly after each other. However, small differences in heart rate, inotropy, and lusitropy might exist between the bSSFP images and the flow data which could affect the results. There was a difference between the relative volume of the DEF and RI flow components, especially during stress. It is not likely that any left sided valve leakage contributed significantly to this difference as the regurgitations were minor. Any body movement between the bSSFP images and flow data was manually corrected in-plane with a registration algorithm. This registration process was challenging in some datasets acquired during dobutamine. The DEF component is to a large extent located along the anterior interventricular septum in healthy hearts, and thus, is more sensitive to subtle spatial mismatch between bSSFP and flow data compared to the DF component. This may contribute to the fact that the relative volume of DEF decreased at stress compared to resting conditions, whereas the relative volume of RI was unchanged. The DF is the component that is least sensitive to errors in the post-processing, and if errors affect the DF component, these almost always result to an underestimation of the absolute volume of the DF component. In this study, the relative volume of the flow components at baseline are in line with previous findings, while we see an increase in the relative volume of DF under stress. It is therefore unlikely that systematic errors in the post-processing would contribute to any significant change in the absolute volume of the DF component in the stress data. However, systematic error might have affected the absolute volume of the DEF component. The relative volume of the DEF and RI components should be theoretically equal, but the difference at stress is on average 20–13 = 7%. Most likely, this is caused by an underestimation of the absolute volume of the DEF leading to an underestimation in the relative volume of the DEF and consequently some overestimation of the relative volume of the other three components. Even if the relative DF volume would be overestimated with 7% at stress, which is a very unlikely worst-case scenario, there would still be a significant increase in the relative DF volume at stress compared to rest.

## Conclusion

This study demonstrates that it is feasible to compare patterns of 4D flow within the normal human heart at rest and with stress. At higher heart rate, inotropy and lusitropy elicited by dobutamine infusion, the efficiency of the intraventricular blood flow transit improves, as quantified by an increased relative volume and ED KE of the most efficient Direct flow LV volume component. The change in these flow-specific markers may allow a novel assessment of LV function and LV dysfunction over a range of stress.

## Data Availability Statement

The raw data supporting the conclusions of this article can be made available by the authors, upon reasonably request. Requests to access the datasets should be directed to Carl-Johan Carlhäll, carl-johan.carlhall@liu.se.

## Ethics Statement

The studies involving human participants were reviewed and approved by Regional Ethical Review Board in Linköping. The patients/participants provided their written informed consent to participate in this study.

## Author Contributions

AB, TE, and C-JC conceived and designed the study. JE, TE, and C-JC were involved in data collection. JS analyzed the data and prepared the figures. JS, AB, EN, and C-JC interpreted the results. JS and C-JC drafted the manuscript. JE, EN, TE, and AB edited the manuscript critically. All authors read and approved the final manuscript.

## Conflict of Interest

The authors declare that the research was conducted in the absence of any commercial or financial relationships that could be construed as a potential conflict of interest.

## Abbreviations

DF: direct flow
DEF: delayed ejection flow
RI: retained inflow
RV: residual volume
bSSFP: balanced steady-state free precession
KE: Kinetic energy.
